# High-sensitive and rapid detection of *Mycobacterium tuberculosis *infection by IFN-γ release assay among HIV-infected individuals in BCG-vaccinated area

**DOI:** 10.1186/1471-2172-10-31

**Published:** 2009-05-28

**Authors:** Weimin Jiang, Lingyun Shao, Ying Zhang, Shu Zhang, Chengyan Meng, Yunya Xu, Lingli Huang, Yun Wang, Ying Wang, Xinhua Weng, Wenhong Zhang

**Affiliations:** 1 Department of Infectious Diseases, Huashan Hospital, Fudan University, Shanghai 200040, PR China; 2 Department of Molecular Microbiology & Immunology, Bloomberg School of Public Health, Johns Hopkins University, Baltimore, MD 21205, USA; 3 Department of Infectious Diseases, Honghe No.1 People's Hospital, Yunnan Province 661100, PR China

## Abstract

**Background:**

An accurate test for *Mycobacterium tuberculosis *infection is urgently needed in immunosuppressed populations. The aim of this study was to investigate the diagnostic power of enzyme-linked immunospot (ELISPOT)-based IFN-γ release assay in detecting active and latent tuberculosis in HIV-infected population in *bacillus Calmette-Guerin *(BCG)-vaccinated area. A total of 100 HIV-infected individuals including 32 active tuberculosis patients were recruited. An ELISPOT-based IFN-γ release assay, T-SPOT.TB, was used to evaluate the *M. tuberculosis *ESAT-6 and CFP-10 specific IFN-γ response. Tuberculin skin test (TST) was performed for all recruited subjects.

**Results:**

The subjects were divided into group HIV+ATB (HIV-infected individuals with active tuberculosis, n = 32), group HIV+LTB (HIV-infected individuals with positive results of T-SPOT.TB assay, n = 46) and group HIV only (HIV-infected individuals with negative results of T-SPOT.TB assay and without evidence of tuberculosis infection, n = 22). In group HIV+ATB and HIV+LTB, T-SPOT.TB positive rate in subjects with TST <5 mm were 50% (16/32) and 41.3% (19/46), respectively. Individuals in group HIV+ATB and HIV+LTB with CD4+ T cells <500/μl, T-SPOT.TB showed a higher sensitivity than TST (64.5% vs. 22.6% and 62.2% vs. 29.7%, respectively, both *P *< 0.0001). In addition, the sensitivity of T-SPOT.TB assay in group HIV+ATB increased to >85% in patients with TB treatment for less than 1 month and CD4+ T cells ≥200/μl, while for patients treated for more than 3 months and CD4+ T cells <200/μl, the sensitivity was decreased to only 33.3%. Furthermore, the results could be generated by T-SPOT.TB assay within 24 hours, which was more rapid than TST with 48–72 hours.

**Conclusion:**

ELISPOT-based IFN-γ release assay is more sensitive and rapid for the diagnosis of TB infection in Chinese HIV-infected individuals with history of BCG vaccination, and could be an effective tool for guiding preventive treatment with isoniazid in latently infected people and for TB control in China.

## Background

HIV and tuberculosis (TB) are two major infectious killers of adults in the developing world, and approximately 13 million people are infected with both causative organisms. The world health organization (WHO) estimated that 0.7 million HIV-positive cases of the 9.2 million (8% of the total) new cases of TB in 2006 were HIV positive [1]. One-third of the world population harbors *Mycobacterium tuberculosis (M. tuberculosis) *in an asymptomatic, latent form (latent tuberculosis infection, LTBI), about 5–10% of which develop TB disease during their lifetime, mostly within 5 years of infection. However, the risk of developing active TB disease in those that are co-infected with HIV increases to 5–15% annually, as the immune deficiency worsens [1,2].

Detection of latent or recent infection, which represents a reservoir of future tuberculosis cases, offers an opportunity for intensified control [3]. Results of a meta-analysis suggest that isoniazid preventive therapy (IPT) reduces TB incidence by 42% overall, or by 60% among individuals who have positive tuberculin skin test (TST) [4,5]. In HIV-infected people with LTBI, IPT is an underutilized public health strategy. However, the skin reaction to tuberculin purified protein derivative (PPD) is impaired by HIV infection [6,7]. Furthermore, TST has poor specificity in areas where *bacillus Calmette-Guerin *(BCG) is used and where there is high prevalence of environmental mycobacteria [8]. The introduction of interferon (IFN)-γ release assays (IFNGRAs) using immunogenic and specific *M. tuberculosis *antigens (early secreted antigen ESAT-6 and culture filtrate protein 10 kD CFP-10) for immunodiagnosis is therefore a potential advance. Compared with the TST, studies using IFNGRA demonstrate a high sensitivity for active tuberculosis [9-11]. Positive scores in these assays have also been shown in contact studies to equate well with a history of exposure to tuberculosis [12-14]. Two commercial forms of the IFNGRA are now licensed for use in the developed world: the T-SPOT.TB (Oxford Immunotec, Abingdon, UK), which has been developed based on the enzyme-linked immunospot (ELISPOT) assay [15]; and the whole blood-based QuantiFERON-TB Gold (QFT-G; Cellestis, Melbourne, Australia), which uses an enzyme-linked immunosorbent assay (ELISA) to detect IFN-γ released into culture supernatant [16].

A prospective study in immunocompetent individuals showed that T-SPOT.TB and QuantiFERON-TB Gold have higher specificity than TST. Overall, T-SPOT.TB produced significantly more positive results (38%, n = 144) than QuantiFERON-TB Gold (26%, n = 100) (*P *< 0.0001) [17]. Several comparison studies of T-SPOT.TB with TST indicate that T-SPOT.TB appears to be a better way to identify *M. tuberculosis *infection than TST even in a BCG-vaccinated population [18,19]. However, the sensitivity and specificity of T-SPOT.TB in diagnosing *M. tuberculosis *infection including active and latent status in HIV-infected individuals have not been systemically evaluated. Furthermore, studies in TB pandemic countries are rather limited. We detected *M. tuberculosis *infection using TST as well as T-SPOT.TB assay in 102 HIV-infected individuals in Yunnan province in China, a region with high prevalence of HIV infection due to the drug abuse. The aims of this study were to determine the diagnostic power of T-SPOT.TB assay in active and latent *M. tuberculosis *infections in HIV-infected population in comparison with TST and to evaluate the effect of T cell numbers on the sensitivity of the T-SPOT.TB and TST.

## Methods

### Study population

**Figure 1:**
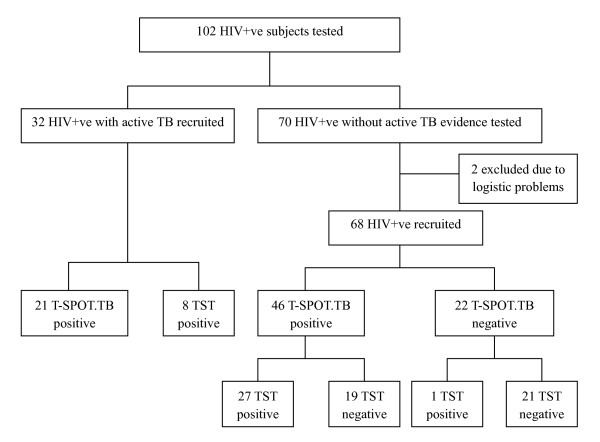
**Flow chart showing numbers recruited in HIV-positive (HIV+ve) individuals with or without active tuberculosis**. TB = tuberculosis; TST = tuberculin skin test; T-SPOT.TB: commercial test IFNGAR from Oxford Immunotec, Abingdon, UK.

One hundred and two HIV-infected individuals from Yunnan province, the HIV and TB pandemic area in China, were included in this study. HIV infection was confirmed by clinical records, routine serum tests (competitive ELISA and Western blotting confirmation) and CD4+ T cell counts. All subjects had a history of newborn BCG vaccination and were received TST using PPD at enrollment (see figure 1). The study subjects were categorized as HIV-infected individuals with active tuberculosis (HIV+ATB), HIV-infected individuals with latent tuberculosis infection (HIV+LTB) and HIV-infected individuals without evidence of tuberculosis infection (HIV only).

### Criteria for determining HIV+ATB and HIV+LTB subjects

Since BCG vaccination is still one of the national routine immunization items and widely implemented in China, we used T-SPOT.TB assay to distinguish BCG vaccination from *M. tuberculosis *infection. The HIV-infected subjects were diagnosed with active TB (ATB) or latent TB (LTB) based on T-SPOT.TB assay and extensive clinical evaluation of tuberculosis, including TB contact history, chest x-ray, sputum smear and culture. The combined T-SPOT.TB assay and extensive clinical evaluation resulted in the classification of three groups of human subjects: HIV+ATB, HIV+LTB and HIV only. Thirty-two patients (22 men and 10 women, 37.0 ± 8.6 years of age, range 26–65 yr) were defined as having ATB (HIV+ATB) by the presence of recent clinical symptoms of TB, positive culture of *M. tuberculosis *and/or positive smear test for acid-fast bacilli (AFB) from sputum and abnormal chest radiograph. 46 (24 men and 22 women, 33.9 ± 6.7 years of age, range 24–45 yr) were diagnosed with latent TB (HIV+LTB) by positive T-SPOT.TB assay and absence of any active tuberculosis, including clinical manifestations of pulmonary and extra-pulmonary tuberculosis as well as abnormal chest radiograph. 22 HIV-infected individuals (9 men and 13 women, 33.7 ± 5.8 years of age, range 22–44 yr) were defined as no *M. tuberculosis *co-infection group (HIV only) based on negative T-SPOT.TB assay and the absence of any clinical evidence of tuberculosis.

### T-SPOT.TB assay

T-SPOT.TB kit (Oxford Immunote Ltd., Oxford, UK), a novel commercial ELISPOT assay to detect IFN-γ release induced by *M. tuberculosis *ESAT-6 and CFP-10, was employed to identify *M. tuberculosis *infection including latent and active *M. tuberculosis *infection. The test result of T-SPOT.TB assay was considered positive if either or both of Panel A (containing peptide antigens derived from ESAT-6) or Panel B (containing peptide antigens derived from CFP-10) had six or more spots than the negative control, and this number was at least two times greater than the number of spots in the negative controls according to the manufacturer's instructions. The spots were read using the ELISPOT plate reader (AID-Gmb-H, Germany). The results were double checked by other lab workers and, if necessary, corrected by manual counting. The laboratory technicians were blinded to the subject identifiers.

### TST

TST was carried out and read by one individual, using the Mantoux technique on the volar surface of a forearm, with five tuberculin units (TU) of tuberculin PPD RT23 (Statens Seruminstitut, Copenhagen, Denmark). Tests were read at 48–72 h, and were measured with a ruler as induration diameters along and across the arm.

### Data analysis

Statistical analysis was done using the non-parametric Mann-Whitney test and Fisher's exact test. Significance was inferred for *P *< 0.05.

## Results

### Baseline characteristics of enrolled subjects according to *M. tuberculosis *infection status

One hundred and two HIV-infected individuals were tested. Thirty-two patients with active TB (ATB) and 68 without ATB evidence were recruited. T-SPOT.TB results of 2 individuals without ATB were not available due to logistic problems (i.e. the persons failed to go to the lab for the test) and were then excluded (Figure 1). Baseline information for 100 recruited subjects is shown in Table 1.

**Table 1:** Baseline data for all the recruited subjects screened with T-SPOT.TB assay and TST

	HIV+ATB	HIV+LTB	HIV only
Patient No.	32	46	22
Age (years) median (SD)	37.0 (8.6)	33.9 (6.7)	33.7 (5.8)
Male/Female	22/10	26/22	9/13
BCG vaccination history	Yes	Yes	Yes
CD4+ T cell counts, n (%)			
<200/μl	21 (65.6)	4 (8.7)	5 (22.7)
200–500/μl	10 (31.3)	19 (41.3)	9 (40.9)
>500/μl	1 (3.1)	23 (50.0)	8 (36.4)
Anti-TB treatment, n (%)			
<1 mo	18 (56.3)	No	No
1–3 mo	9 (28.1)		
>3 mo	5 (15.6)		
HAART, n (%)	20 (62.5)	4 (8.7)	2 (9.1)

Thirty-two HIV positive patients with ATB were assigned into group HIV+ATB. Nineteen of 32 patients had been receiving TB treatment with the longest duration of 12 months, while the remaining 13 patients were not treated with anti-TB drugs upon enrollment. In group HIV+ATB, 21 (65.6%) patients were T-SPOT.TB positive and 8 (25%) were TST positive (*P *= 0.013) (Figure 1). LTBI was diagnosed with T-SPOT.TB in individuals without ATB evidence. Forty-six of 68 were T-SPOT.TB positive and assigned into group HIV+LTB. The remaining 22 with negative T-SPOT.TB test were named as group HIV only (Figure 1). Based on CD4+ T cell count, the subjects in these 3 groups were divided into 3 subgroups: CD4+ T cells <200/μl, 200–500/μl and >500/μl. At recruitment, TST results were read and recorded individually by one observer.

### Comparison of T-SPOT.TB with TST results in groups HIV+ATB and HIV+LTB

**Figure 2:**
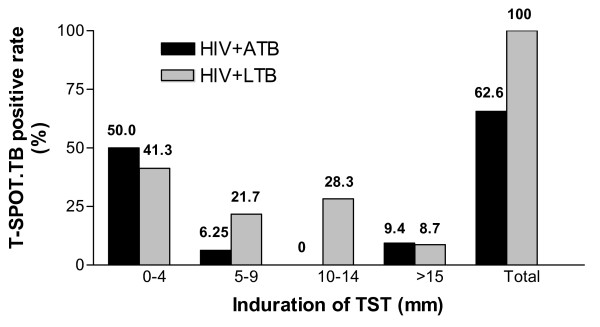
**T-SPOT.TB positive rate in group HIV+ATB and HIV+LTB stratified by indurations of TST (mm)**. The black column represents the T-SPOT.TB positive rate of patients in HIV+ATB group, and the gray column represents the T-SPOT.TB positive rate of patients in HIV+LTB group. The T-SPOT.TB positive portion with TST 0–4 mm was misdiagnosed by TST.

We divided the subjects in both groups into different subgroups respectively based on the TST diameter: 0–4 mm, 5–9 mm, 10–14 mm and >15 mm (Figure 2). In group HIV+ATB, 21 of 32 (65.6%) were T-SPOT.TB positive, whereas TST positive rate was only 15.6% (5/32) (*P *< 0.005). Among 11 T-SPOT.TB negative patients, 7 were with CD4+ T cells <200/μl and 4 with CD4+ T cells 200–500/μl. In 24 TST negative patients, 16 had CD4+ T cells <200/μl and 8 with CD4+ T cells 200–500/μl.

When 5 mm induration was used as the cutoff for TST, 16 of 32 (50%) HIV+ATB patients with negative TST (induration ≤4 mm) were positive in T-SPOT.TB assay, while among HIV+LTB persons, 19/46 (41.3%) with TST ≤4 mm were T-SPOT.TB positive. Therefore, this portion was misdiagnosed by TST in HIV-infected individuals. In subjects with TST >5 mm, T-SPOT.TB assay did not demonstrate any advantage of diagnosis compared with TST.

### Effect of CD4+ T cell count on T-SPOT.TB assay in HIV infected individuals

**Figure 3:**
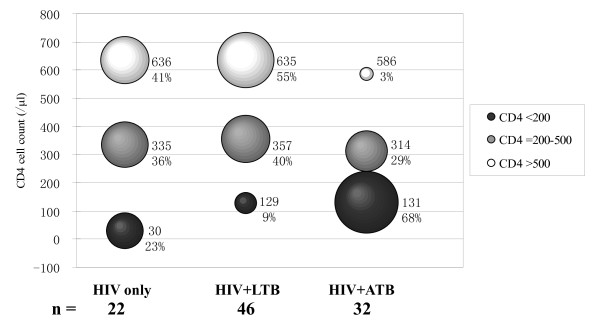
**The distributions of subjects with different CD4+ T cell counts in HIV only, HIV+LTB, and HIV+ATB groups**. The black bubbles represent CD4+ T cells <200/μl, the gray bubbles represent CD4+ T cells 200–500/μL, and the white bubbles represent CD4+ T cells >500/μl. The sizes of the bubbles represent the percentage of patients with appropriate CD4+ T cell counts.

CD4+ T cell count which represents the immune status or HIV/AIDS progression of the patients is a key factor in HIV infected persons that could affect the TB diagnosis. In Figure 3, the bubbles showed the distribution of CD4+ T cell count in different groups. In HIV only group, the individuals with CD4+ T cells <200 μl, 200–500/μl and >500/μl distributed relatively evenly. However in the other two groups, the patients in different subgroups based on CD4+ T cell count distributed reversely. CD4+ T cell count in the majority of the patients in HIV+LTB group was 500/μl, while most patients in group HIV+ATB were CD4+ T cells <200/μl, which possibly indicated the role of CD4+ T cells in controlling TB activation and the high risk of progression from latent infection to active tuberculosis.

**Figure 4:**
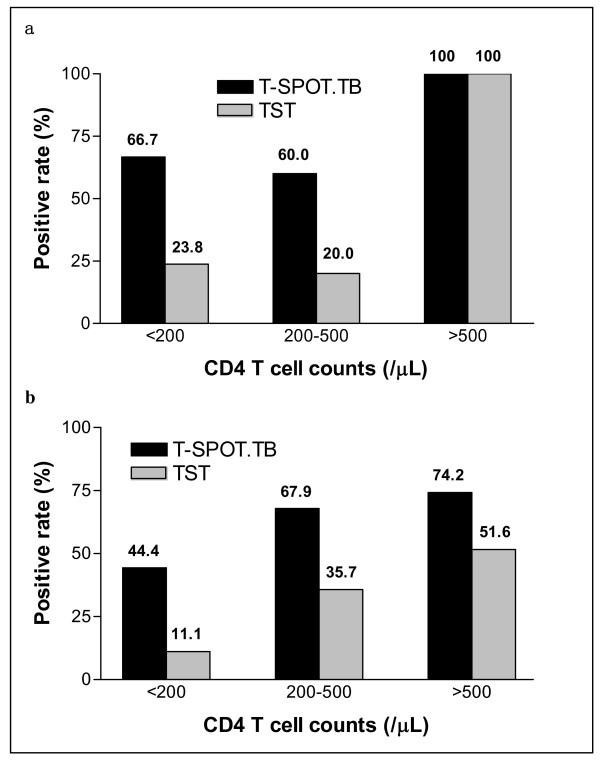
**Positive rates of HIV+ individuals to ESAT-6/CFP-10 T-SPOT.TB and to TST, by CD4+ T cell count**. The black column represents the T-SPOT.TB positive rate, and the gray column represents TST positive rate. (a) Patients with active TB (group HIV+ATB). In subgroups of CD4+ T cells <200/μl and 200–500/μl, both *P *< 0.0001; (b) Individuals without active TB evidence (group HIV+LTB and HIV only). In subgroups of CD4+ T cells <200/μl and 200–500/μl, both *P *< 0.0001.

In addition, we compared T-SPOT.TB with TST results according to different CD4+ T cell counts both in patients with ATB and persons without ATB (Figure 4a, b). In HIV+ATB patients with CD4+ T cells <200/μl and 200–500/μl, T-SPOT.TB showed a higher sensitivity than TST (both *P *< 0.0001). Since there was only one patient with CD4+ T cells >500/μl who was both T-SPOT.TB and TST positive, it was not compared and included in this subgroup, which is similar to individuals without ATB. In subgroup of CD4+ T cells <200/μl and 200–500/μl, T-SPOT.TB appeared to be a more sensitive assay to diagnose LTB than TST (both *P *< 0.0001). However, no significant difference was found between the two tests in individuals with CD4+ T cells >500/μl. These results strongly suggest that T-SPOT.TB assay markedly increased TB diagnostic power especially in subjects with CD4+ T cells <500/μl.

### Combined impact of TB treatment and total CD4+ T cells on T-SPOT.TB assay

**Figure 5:**
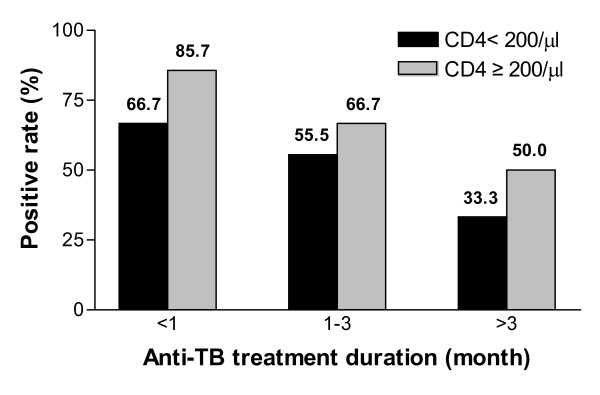
**T-SPOT.TB positive rates in patients with active TB by different durations of TB treatment and CD4+ T cell counts**. The black column represents the T-SPOT.TB positive rate of patients with CD4+ T cells <200/μl, and the gray column represents that of patients with CD4+ T cells ≥200/μl. There are decreasing tendency of T-SPOT.TB positive rate with increasing treatment duration in both CD4+ T cells ≥200/μl and CD4+ T cells <200/μl subgroups. But *P *> 0.05 between subgroups of different treatment durations and subgroups of different CD4+ T cell counts.

The patients in group HIV+ATB were divided into 3 subgroups according to different durations of TB treatment: <1 month, 1–3 months and > 3 months. IFN-γ responses to either ESAT-6 or CFP-10 in patients in 3 subgroups were examined. There was a decreasing tendency of IFN-γ response with increasing treatment duration in both CD4+ T cells ≥200/μl and <200/ul subgroups. However, no significant difference was found. Due to the impact of TB treatment duration and the total CD4+ T cells on the positive rate of T-SPOT.TB assay in HIV combined active tuberculosis infection, we found that the sensitivity of T-SPOT.TB assay could increase to >85% in the patients with TB treatment for less than 1 month and CD4+ T cells ≥200/μl, while the sensitivity for patients treated for more than 3 months and CD4+ T cells < 200/μl was only 33.3% (Figure 5).

## Discussion

TB infection control measures need to be implemented urgently in high-burden countries, which could have important global implications. First of all, there is a clear need a rapid and useful tool to identify TB infection including both active and latent TB accurately. In China, TST has low specificity due to mandatory BCG vaccination, and also low sensitivity especially in immunosuppressed (i.e. HIV-1 infection) population. The recently available *M. tuberculosis *antigen-specific IFNGRA thus represents a significant advancement in the field of TB diagnostics. Studies confirmed that IFNGRA was more sensitive and specific than TST and independent of CD4+ T cell count in moderate to advanced HIV infected humans. Furthermore, the results could be generated by T-SPOT.TB assay within 24 hours, which was more rapid than TST with 48–72 hours. Although T-SPOT.TB assay was more sensitive than TST, the positive rate of T-SPOT.TB assay was decreased to only 44.4% in HIV patients with CD4+ T cells <200/μl, comparing 74.2% and 67.9% in HIV patients with CD4+ T cells >500/μl and 200–500/μl, respectively. Therefore, in severely immunosuppressed individuals, IFNGRA may be impaired by T-cell anergy [20-22].

There are mutiple factors that may affect IFNGRA, such as TB treatment and immunosuppression status. Chee et al concluded that anti-TB treatment had different effect on T-cell response to ESAT-6 and CFP-10 as measured by T-SPOT.TB [23]. Wilkins et al found that preventive treatment of LTBI resulted in 1.8 fold average increase in the number of IFN-γ-producing T cells within 26 ± 4 days, followed by a decrease by the end of the treatment period (82 ± 6 days) [3]. Therefore, we hypothesized that T-SPOT.TB could serve as an effective indicator of the TB therapeutic efficacy. In our study, patients in HIV+ATB group were classified into subgroups according to different treatment durations: <1 mo, 1–3 mo and >3 mo. We found that there was a decreasing tendency of positive T-SPOT.TB assay with increasing treatment duration. Unfortunately, the difference did not appear to be statistically significant, perhaps due to the insufficient number of the recruited subjects. Thus, a larger cohort study is needed to further determine this in the future study.

To our knowledge, this is the first study in which T-SPOT.TB and TST were compared in ATB and LTB among HIV-infected humans in BCG-vaccinated area. Recently, Mandalakas and his colleagues have found that T-SPOT.TB may have improved sensitivity for detection of *M. tuberculosis *infection in HIV-infected individuals compared to the QTF and the TST [24]. However, their study was conducted in a setting highly endemic for TB in South Africa, but not in routinely BCG-vaccinated area. Therefore, the T-SPOT.TB assay appears more sensitive and specific for diagnosing both LTBI in HIV patients with history of BCG vaccination and with confirmed active TB in our study, indicating that the ELISPOT-based IFNGRA may be a better tool for screening tuberculosis infection in China as well as useful information for further implementation of LTBI preventive treatment.

Analysis in this study further confirmed that ELISPOT-based IFNGRA was much more sensitive in patients with lower CD4+ T cells level (<500/μl) than TST; T-SPOT.TB assay was independent of CD4+ T cell counts as well as history of BCG vaccination. Thus, it is evident that not only active TB but also latent TB could be identified successfully in HIV-infected people, providing solid basis to screen latent TB individuals for implementing TB preventive treatment.

## Conclusion

In summary, ELISPOT-based IFNGRA is more sensitive and specific than TST for TB diagnosis in Chinese HIV-infected individuals and could be an effective tool for guiding preventive treatment with isoniazid in latently infected people and for TB control in China. A larger prospective cohort study is needed to further confirm our findings in the future.

## Authors' contributions

WJ carried out the patient collection and clinical data analysis, participated in the tuberculin skin test and helped draft the manuscript. LS carried out the immunoassays, performed the statistical analysis and drafted the manuscript. WZ carried out the design of the study, performed the statistical analysis and helped draft the manuscript. YZ helped to draft the manuscript. XW participated in the design of the study. YX and YW participated in the patient collection and clinical data analysis. SZ, CM, LH and YW participated in the immunoassays and the statistical analysis. All authors read and approved the final manuscript.
